# Characterisation of Matrix-Bound Nanovesicles (MBVs) Isolated from Decellularised Bovine Pericardium: New Frontiers in Regenerative Medicine

**DOI:** 10.3390/ijms25020740

**Published:** 2024-01-06

**Authors:** Dalila Di Francesco, Carolina Di Varsavia, Simona Casarella, Elena Donetti, Marcello Manfredi, Diego Mantovani, Francesca Boccafoschi

**Affiliations:** 1 Department of Health Sciences, University of Piemonte Orientale “A. Avogadro”, 28100 Novara, Italy; dalila.di-francesco.1@ulaval.ca (D.D.F.); 20023413@studenti.uniupo.it (C.D.V.); simona.casarella@uniupo.it (S.C.); 2 Laboratory for Biomaterials and Bioengineering, CRC-I, Department of Min-Met-Materials Engineering, University Hospital Research Center, Regenerative Medicine, Laval University, Quebec City, QC G1V 0A6, Canada; diego.mantovani@gmn.ulaval.ca; 3 Department of Biomedical Sciences for Health, University of Milan, 20133 Milan, Italy; elena.donetti@unimi.it; 4 Department of Translational Medicine, Centre of Excellence in Aging Sciences, University of Piemonte Orientale “A. Avogadro”, 28100 Novara, Italy; marcello.manfredi@uniupo.it; 5 Center for Translational Research on Autoimmune and Allergic Diseases, Department of Translational Medicine, University of Piemonte Orientale “A. Avogadro”, 28100 Novara, Italy

**Keywords:** matrix-bound nanovesicles (MBVs), extracellular vesicles, decellularised extracellular matrix, extracellular matrix biomaterials

## Abstract

Matrix-bound nanovesicles (MBVs) are a recently discovered type of extracellular vesicles (EVs), and they are characterised by a strong adhesion to extracellular matrix structural proteins (ECM) and ECM-derived biomaterials. MBVs contain a highly bioactive and tissue-specific cargo that recapitulates the biological activity of the source ECM. The rich content of MBVs has shown to be capable of potent cell signalling and of modulating the immune system, thus the raising interest for their application in regenerative medicine. Given the tissue-specificity and the youthfulness of research on MBVs, until now they have only been isolated from a few ECM sources. Therefore, the objective of this research was to isolate and identify the presence of MBVs in decellularised bovine pericardium ECM and to characterise their protein content, which is expected to play a major role in their biological potential. The results showed that nanovesicles, corresponding to the definition of recently described MBVs, could be isolated from decellularised bovine pericardium ECM. Moreover, these MBVs were composed of numerous proteins and cytokines, thus preserving a highly potential biological effect. Overall, this research shows that bovine pericardium MBVs show a rich and tissue-specific biological potential.

## 1. Introduction

Decellularised extracellular matrix (dECM) biomaterials have long been of interest for regenerative medicine and tissue engineering applications because of their ability to closely mimic the physiological environment of tissues. These biomaterials can be derived from different biological and tissue sources through the process of decellularisation, which ensures the removal of the cellular component while mainly preserving extracellular matrix (ECM) composition [1,2]. dECM biomaterial products have already successfully demonstrated their bioactivity as providers of a rich and stimuli-charged microenvironment for cells, being able to influence cellular behaviour and even modulate the immune system’s response, thus representing optimal candidates from a regenerative point of view [3,4]. Because of this, effective results have been reported using dECM biomaterials for wound healing [5], nervous system repair [6], liver fibrosis treatment [7], myocardial infarction treatments [8], corneal repair [9], cartilage repair [10], tendon repair [11], hernias [12], and other applications [1,13]. To date, the clear mechanism through which dECM biomaterials are able to guide cellular processes and the immune system to promote tissue regeneration, especially in pathological cases, is not completely clear. The generally acknowledged hypotheses remain that this superior biological performance is related to the complex composition of dECM, which retains major ECM structural components such as collagens, glycoproteins, and proteoglycans [14], coupled with dECM biomaterials’ ability to not simply evade the immune system but interact with it [15,16,17]. This latter ability is known as immunomodulation, meaning the capability to drive the phenotype of immune cells from an inflammatory to a regenerative status; and this property appears to be intrinsic in dECM biomaterials [4,18]. In recent years, additional information has been discovered on the biological potential of dECM-derived biomaterials, and different studies have shown that many of these regenerative and immunomodulatory processes are driven by the exposition of dECM’s bioactive degradation products. These products are often obtained by the degradation of the dECM matrix, which happens either because of the decellularisation process, because of enzymatic degradation, and/or due to the solubilisation methods used to obtain dECM biomaterials such as hydrogels or bioinks [15]. These degradations allow the exposure of highly bioactive molecules which are normally entrapped in the matrix, like peptide fragments, known as matrikines, chemokines, cytokines, and matrix-bound nanovesicles (MBVs) [19,20,21,22,23]. Among these products, MBVs have recently been discovered and identified as major providers of regenerative and immunomodulatory potential in dECM biomaterials. 

MBVs are a subclass of extracellular vesicles (EVs), which range in size from 20 to 400 nm. They are distinguished from other EVs because they are found tightly adhering to the collagen network of ECM and because they have tissue-specific surface markers and cargo content, which includes lipids, proteins, signalling cytokines and chemokines, and nucleic acids [23,24,25,26]. These contents seem to greatly contribute to the immunomodulatory potential of dECM biomaterials. In different studies, MBVs, even when isolated from different tissue sources, have shown the ability to recapitulate the immunomodulatory and regenerative potential of dECM, and have been successfully used both in vitro and in vivo for different regenerative medicine applications, suggesting their vast potential for biomedical applications [27,28,29,30,31,32,33,34]. Even though MBVs seem to be a ubiquitous component of the ECM, maintained even after the tissue decellularisation process, to date, they have only been isolated from small intestine submucosa, urinary bladder matrix, dermis, brain, oesophagus, heart, muscle, liver, ovary, pancreas, and tendons [23,24,25,35]. The research characterising these MBVs has shown that there are slight differences in the cargo, surface markers, and, thus, biological potential, of each tissue-specific MBV. Moreover, because MBVs are found adhering to the ECM, unlike other EVs which are found in body fluids, their isolation also becomes more challenging and needs to be standardised with respect to each tissue. In fact, while for other EVs, standard extraction methods have been well described, and include size exclusion chromatography, density gradient centrifugation, and differential centrifugation, MBVs need tougher methods to be harvested [24,36]. First off, because MBVs are attached to the ECM, the ECM-derived material first needs to undergo a decellularisation process to obtain dECM, which can include detergent-based and enzymatic-based decellularisation methods. Secondly, the dECM needs to be further solubilised in order to detach the MBVs from the collagen matrix. Once these steps are performed, the MBVs can be isolated from the solubilised dECM by the protocols previously reported for EVs. This implies that MBVs experience several mechanical, chemical, and enzymatic processes before they can be harvested, each step possibly altering the properties, quality, and yield of the obtained MBVs [24,37]. It is therefore important to understand the presence, optimal harvesting method, and role of MBVs in different dECM biomaterials to be able to fully exploit the potential of these materials and properly apply them in a regenerative context.

In this challenging context, the objective of this research was to evaluate the presence of MBVs in decellularised bovine pericardium (dBP). dBP is a renowned biomaterial, for which decellularisation protocols have already been effectively standardised, that has both already been translated into clinics, and that has shown excellent regenerative potential in different applications [13,38,39,40,41,42,43,44]. Here, MBVs from dBP were characterised in terms of their tissue-specific physical and chemical properties, and finally, their cytocompatibility was evaluated for future regenerative medicine applications. The results obtained showed that MBVs can be isolated from dBP and present a plentiful protein cargo, along with a variety of immunomodulatory molecules such as cytokines and chemokines, and demonstrate cytocompatibility.

## 2. Results

### 2.1. Matrix-Bound Nanovesicles from Decellularised Bovine Pericardium

**Figure 1:**
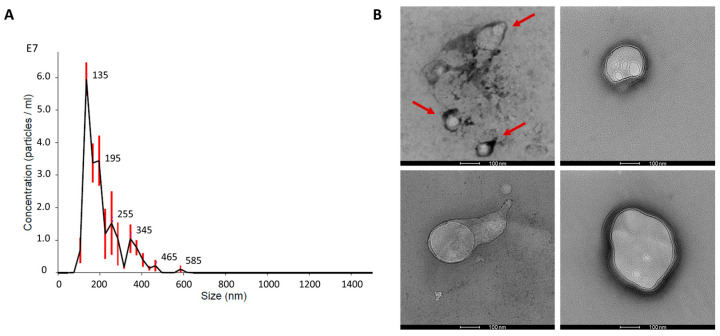
(**A**): Representative concentration-size distribution of NTA analysis on MBVs is shown. Red bars indicate ± standard error of the mean. (**B**): Representative TEM images of MBVs fixed on carbon-coated grids. Red arrows highlight MBVs aggregated. Scale bar 100 nm.

The results of nanoparticle tracking assay (NTA) and transmission electron microscopy (TEM) evidenced the presence of nanovesicles isolated from dBP by enzymatic isolation and differential centrifugation. NTA was performed on MBVs resuspended in particle-free phosphate-buffered saline (PBS) and showed that a mean of 1.9 × 10^8^ ± 0.22 × 10^8^ MBVs/mL, starting from 100 mg of dry weight dBP, could be obtained. The MBVs showed a mode size of 142 ± 1.5 nm. A representative curve of one NTA analysis out of the triplicate is shown in Figure 1A, indicating an MBV size peak ranging from 100 to 200 nm, correlated to the size of MBVs found in the literature. MBVs’ size range was also confirmed by TEM analysis (Figure 1B), in which MBVs characterised by a generally rounded lipidic membrane, stained in black by osmium tetroxide, were found. Some MBVs were observed aggregated to each other (evidenced by red arrows in Figure 1B). Furthermore, most MBVs displayed additional roundish lipidic structures inside the vesicles.

### 2.2. Matrix-Bound Nanovesicles Protein Content

#### 2.2.1. SDS-PAGE and Silver Staining

**Figure 2:**
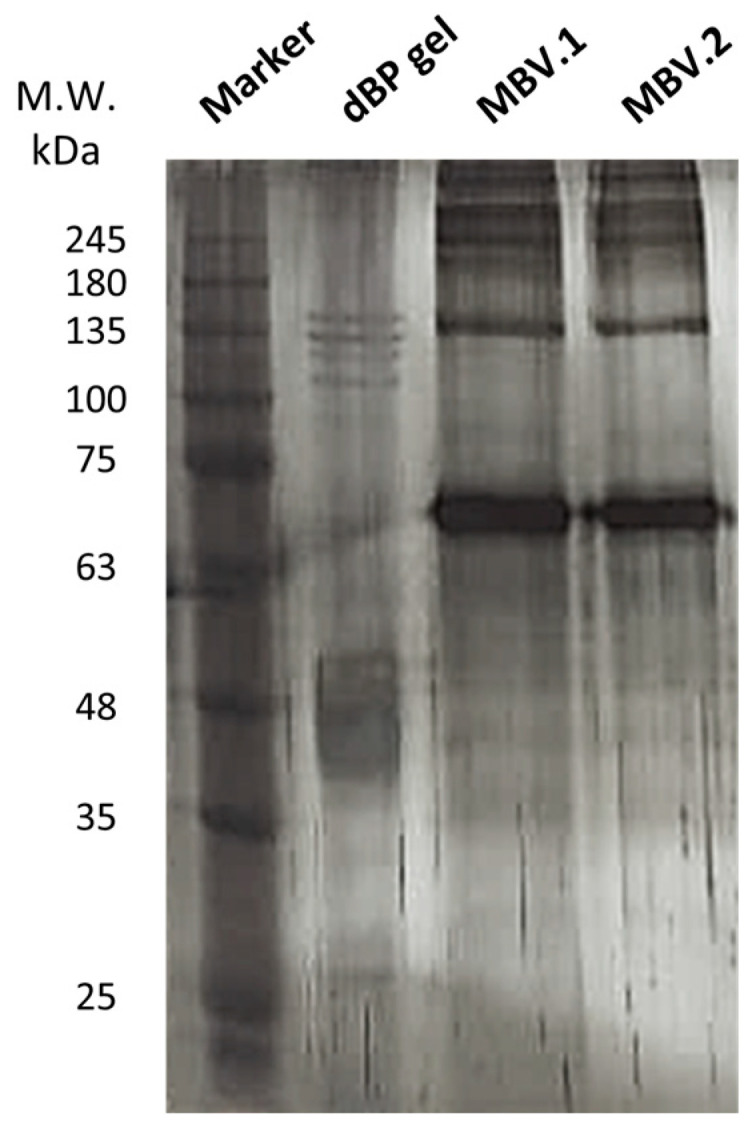
Silver stain on SDS-PAGE. Samples were loaded as follows, left to right: protein marker, dBP gel, MBV.1, MBV.2.

Silver staining was performed on an sodium dodecyl sulfate–polyacrylamide gel electrophoresis (SDS-PAGE) of MBV proteins isolated from different bovine pericardium batches (MBV.1 and MBV.2) and proteins extracted from dBP gel; a representative image of experimental triplicates is shown in Figure 2. The dBP gel was obtained through a process of dBP solubilisation which maintained the protein content of the dBP source matrix containing MBVs, while with the extraction of MBVs, only the proteins contained in the nanovesicles should be retained. The results show the presence of different protein profiles, and in both conditions (dBP gel and MBVs), a variety of proteins ranging from >245 to >25 kDa were observed. Many of the protein bands found in the dBP gel were not observed in the MBV samples. However, some common protein bands can be observed, such as at 135 kDa, ~70 kDa, and ~48 kDa, which were significantly more expressed in the MBV samples. Moreover, the protein profiles in MBV.1 and MBV.2 were highly comparable.

#### 2.2.2. Cytokine Antibody Array

**Figure 3:**
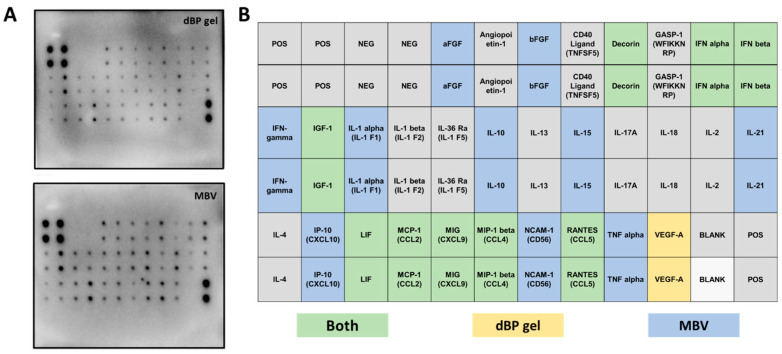
(**A**): Representative antibody array membranes of dBP gel and MBV samples of experimental triplicates using different MBV batches. (**B**): Table showing the order of the cytokine spots on the antibody array membrane. Highlighted in green are cytokines found to be expressed in both dBP gel and MBVs; in yellow, cytokines expressed in dBP gel only; in blue, cytokines expressed in MBVs only; and in grey the cytokines that were not expressed in any of the samples.

The results from the cytokine assay evidenced different cytokines and chemokines contained in MBVs. The detected molecules showed spots on the array membranes, with the spots’ density correlating with the quantity of the detected molecule. Representative membrane images (from experimental triplicates) of cytokine content in dBP gel and MBVs are shown in Figure 3A. Twenty different cytokines were highlighted in the samples (Figure 3B); in particular, acidic and basic fibroblast growth factors (aFGF and bFGF), interferon (IFN) gamma, interleukin (IL) 1 alpha, IL-10, IL-15, IL-21, IFN gamma-induced protein 10 (IP-10), neural cell adhesion molecule (NCAM) 1, and tumour necrosis factor (TNF) alpha were found in higher quantities in MBVs (blue in Figure 3B), while decorin, IFN alpha and beta, insulin-like growth factor (IGF) 1, leukaemia inhibitory factor (LIF), monocyte chemoattractant protein (MCP) 1, monokine induced by IFN-gamma (MIG), macrophage inflammatory protein (MIP) 1 beta, and chemokine ligand 5 (RANTES) were found to be expressed both in MBVs and dBP gel samples (green in Figure 3B). Finally, only vascular endothelial growth factor (VEGF) A was found in the dBP gel and not in MBVs (yellow in Figure 3B).

#### 2.2.3. Protein Mass Spectrometry

Mass spectrometry results showed the protein profile of MBVs isolated from two different pericardium batches (MBV.1 and MBV.2). Table 1 shows the proteins found in both batches of MBVs, of which 45 different proteins were represented in MBV.1 and 23 in MBV.2. Of these, 18 different proteins were common for both batches. In particular, collagen type I, albumin, vimentin, annexin A2, heat shock protein 70 kDa 1-like, complement component C6, tubulin, actin, asporin, histone H4, and prolargin were found in both samples. Glycoproteins (fibulin-5, fibrillin, fibronectin, alpha-2-HS-glycoprotein) and proteoglycans (mimecan and decorin) were also present.

### 2.3. Cytocompatibility

**Figure 4:**
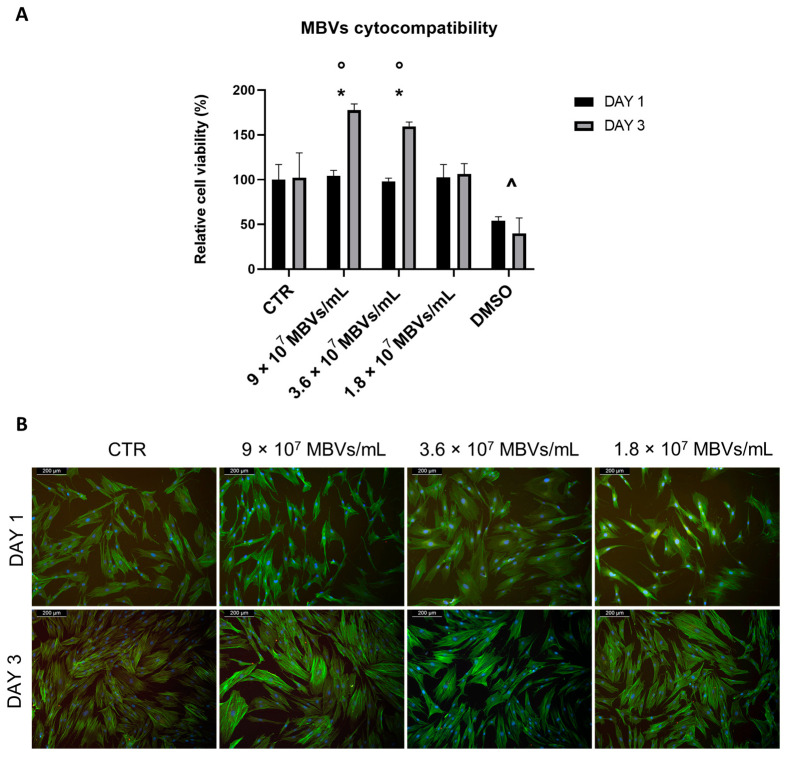
(**A**): MTT viability assay on NHDF cells treated with different concentrations of MBVs for 1 and 3 days. Data are indicated as means ± S.D. of triplicate. *: day 1 vs. day 3; ◦: MBVs vs. CTR day 3; ^ DMSO vs. all conditions, *p* < 0.05. (**B**): Phalloidin-DAPI immunofluorescence staining on NHDF cells treated with different concentrations of MBVs for 1 and 3 days. Images are representative of experimental triplicates.

To test the cytocompatibility of MBVs at different concentrations with normal human dermal fibroblast (NHDF) cells, Thiazolyl Blue Tetrazolium Bromide (MTT) viability assay and phalloidin-4′,6-diamidino-2-phenylindole (DAPI) immunofluorescence staining were performed. Figure 4A shows the results of the viability assay, in which no statistical differences were shown between control (CTR) and cells treated with different concentrations of MBVs on day 1. While in the CTR the cells did not show a significant increase in viability between day 1 and day 3, cells treated with concentrations of 9 × 10^7^ MBVs/mL and 3.6 × 10^7^ MBVs/mL showed a statistically significant increase in cell viability between day 1 and day 3. Finally, these MBV-treated conditions showed significantly higher cell viability at day 3 when compared to the respective CTR, while the 1.8 × 10^7^ MBVs/mL condition, although not showing any cell toxicity, did not increase cell viability. DMSO was used as a control for cell death, and showed statistically significant decreases in cell viability both at days 1 and 3 compared to cell viability across all other conditions. These results were confirmed by immunofluorescence (Figure 4B), where cell morphology analysed with phalloidin-DAPI staining was comparable between MBV groups and CTR. Overall, MBV at different concentrations did not induce cell toxicity or alterations in NHDF cell morphology after 3 days of treatment.

## 3. Discussion

In this work, the presence of matrix-bound nanovesicles (MBVs) in decellularised bovine pericardium (dBP), their physical and chemical properties, and their cytocompatibility were evaluated. 

The results from NTA and TEM analyses showed that nanovesicles, ranging in size from 100 to 200 nm and characterised by a lipidic membrane, were isolated from dBP. These results confirm the presence of MBVs in dBP, a subclass of extracellular vesicles (EVs), distinguished as adhering to the ECM, while EVs were believed to be present solely in body fluids [24]. The size range and structure of MBVs from the TEM results herein reported correspond to MBVs from different tissues described in the literature [23,25,35]. Moreover, the nanovesicle structure also showed additional vesicular internal structures, as observed also for other EVs [45]. These MBVs were isolated from a decellularised tissue, and thus their presence proves their ability to remain attached to the collagen matrix even after tissue decellularisation, and that they are only released after specific enzymatic degradation. Previous works, such as those of Quijano et al., have evaluated different methods of isolating MBVs from dECM [24,37], and these works consider the essential use of initial enzymatic degradation. This is because a tougher extraction method, in comparison to the classic non-enzymatic methods used to isolate other EVs, is needed to obtain MBVs and detach them from the matrix [23]. Therefore, extraction of MBVs from dBP was performed using collagenase degradation coupled with gradient centrifugation. This method resulted in an average of 1.9 × 10^8^ ± 0.22 × 10^8^ MBVs/mL from 100 mg of dry weight dBP, a slightly lower concentration than others reported in the literature, indicating a tissue-dependent and method-dependent yield [37]. Nonetheless, our results prove the occurrence of this newly described subclass of EVs in yet another decellularised tissue. This implies that an additional signalling structure is present, not only in the ECM, but also in dECM-derived biomaterials, and that are released only after enzymatic degradation of the matrix and/or biomaterial [24,46]. 

EVs are widely known for their cell signalling potency, and as research interest in their potential grew, discoveries were made on their major paracrine roles, both physiologically and pathologically, in angiogenesis, immunomodulation, cell proliferation and differentiation, and tissue repair [47,48,49]. This potential comes from their highly bioactive cargo, which has been well studied in recent years for known EVs. In the case of the MBVs, because of their recent discovery, less is known about their cargo. While more work has been performed to understand the nucleic acid and lipidic content of MBVs, in-depth characterisation of the protein content has not yet been reported [24,25,26]. Given that the cargo of MBVs is known to be tissue-specific and to confer the bioactive role to the source dECM material, the characterisation of their protein and cytokine content is essential to elucidate their cellular signalling potential [24,25]. A content comparison was made towards a dBP gel obtained from dBP through a process of enzymatic solubilisation, representing the parent dECM material of MBVs. Thus, both MBVs and dBP gel are obtained through a similar method, with the difference that MBVs are then isolated from the dECM through differential centrifugation, while the dBP gel retains matrix components, including the MBVs. The silver stain performed on the SDS-PAGE evidenced a complex protein cargo in all samples. The protein signatures of different MBV batches (MBV.1 and MBV.2) were shown to be comparable, for which broad protein content reproducibility of MBVs isolated from different pericardium batches can be speculated. When comparing the protein content of MBVs to the dBP gel, slight differences can be noted. The main bands in the MBV samples were also found, albeit in lower expression, in the dBP gel; this is because MBVs should be present inside the dBP gel. A richer protein profile was shown in the dBP gel, derived from dECM proteins retained in the sample; nevertheless, MBVs showed an abundant protein content which had to be further investigated. The results from the cytokine assay also evidenced a rich bioactive content of 19 different cytokines expressed in MBVs. Among these, 9 cytokines were expressed both in MBVs and in the dBP gel, while 10 others appeared to be only expressed in MBVs, and VEGF-A was expressed only in the dBP gel. The variety of cytokines, chemokines, and growth factors observed confirm the bioactivity of MBVs, as these molecules function as immunomodulators. Among growth factors and hormones, acidic and basic FGFs were expressed, which are both involved in tissue repair and angiogenesis [50,51], along with IGF-1, a well-known regulator of cellular and metabolic functions that also has an anti-inflammatory effect [52,53]. The proteoglycan decorin was found, which, in addition to playing a major role in ECM integrity, has immunomodulatory effects [54,55]. Interferons alpha, beta, and gamma were expressed, which play pro-inflammatory, anti-inflammatory, and anti-viral roles [56,57]. Other potent activators of the immune system were found, including TNF-alpha and a diversity of interleukins, like IL-1 alpha, IL-15, and IL-21, or immunosuppressive ones, such as IL-10 [56,58,59,60]. Different chemoattractant chemokines were expressed, like MIP-1 beta, MIG, RANTES, MCP-1, and IP-10 [61,62]. Multifunctional glycoprotein NCAM-1 was also present, which plays a role in cell–cell interactions and cell–matrix interactions, immune system surveillance, and nervous system development [63,64]. Overall, these findings further explain the cell-signalling properties and immunomodulatory potential of MBVs which were already described in the literature. Moreover, the tissue-specificity of the cytokine cargo was also confirmed. Turner et al. reported that, although MBVs isolated from different tissues expressed different levels and types of cytokines, some common properties were shown in all MBVs. For example, their work highlighted the baseline expression of angiopoietin-1 or the low expression of IFN-alpha in MBVs isolated from different tissues [25], while in our work, MBVs isolated from dBP showed a contrary trend, as angiopoietin-1 is not expressed and INF-alpha is, validating the differences in tissue-specific cargo.

The results from mass spectrometry highlight the role MBVs play in the transport of bioactive proteins. The presence of a variety of 18 proteins, shared in two different batches of MBVs, was discovered. Most of these are proteins that are usually secreted in the ECM and that play both structural and non-structural roles, while others are cytoplasmatic proteins. Among the ECM-secreted proteins were collagen type I, which can be secreted in extracellular vesicles for cell signalling between cells [65], and elastic fibre proteins such as fibrillin-1 and fibulin-5 [66]. Mimecan was also present in MBVs, which is a classic component of bovine tissues with growth factor activity and involved in ECM assembly [67]. Other components highlighted are fibronectin, which was shown to play a role in many EV–cellular interactions [68] and have anti-fibrotic activity in tissue repair [69,70], and annexin 2, which is normally found in mineralising tissues and is involved in EV protein trafficking [71]. The presence of decorin was also confirmed. The cellular cytoplasmatic proteins were vimentin, which can be found extracellularly when transported by exosomes [72], tubulin, actin, and histone H4, which has also been previously shown extracellularly in vesicles [73]. This outcome shows a majority of ECM-related proteins, indicating that MBVs are secreted during ECM-involved processes, such as ECM deposition or remodelling, and a different biogenesis from other EV subtypes is hypothesised [24,26]. The results also showed differences in proteins found in the two MBV batches. Because the process of dBP decellularisation is standardised, we argue that the differences come from the well-known batch-to-batch variation in biomaterials derived from animal origin. Moreover, the main differences are noted in a loss of mainly cellular-related peptides, such as enzymes, while there is no loss of ECM-related proteins, suggesting that the intrinsic bioactivity is not lost between batches [2,74,75,76]. Most interestingly, these results not only elucidate the bioactive mechanisms of the ECM’s role in paracrine signalling, but further expand the interest in dECM biomaterials. Herein, we proved that dBP contains signalling factors that are protected by additional lipidic membranes, tightly bound to the biomaterials’ matrix, and that are only released upon the material’s enzymatic degradation. Considering that it is of common interest to design and engineer biomaterials that contain and release such immunomodulatory and regenerative molecules, the knowledge of naturally occurring materials with these properties opens the possibility of their applications in regenerative medicine [49,77,78]. 

Lastly, the cytocompatibility of different MBV concentrations was tested on normal human dermal fibroblasts (NHDFs). The previous literature reported no cytotoxic effects of MBVs even at high concentrations of ~10^11^ MBVs/mL [28]; however, lower concentrations able to produce a biological effect in terms of cellular viability were chosen. The outcomes of the MTT viability assay showed that even at the highest concentration (9 × 10^7^ MBVs/mL) given to cells, no cytotoxic effects were seen. MBV concentrations of 9 × 10^7^ MBVs/mL and 3.6 × 10^7^ MBVs/mL induced a significant increase in cell viability from day 1 to day 3 of cell culture, while also displaying a higher cell viability at day 3 when compared to CTR. The lowest concentration of MBVs used showed no enhancement with respect to cell viability. Thus, we can hypothesise that MVBs at adequate concentrations allow and enhance cell viability through a richer milieu, as proved by the bioactive molecules found in their cargo. The findings of MBV cytocompatibility were also confirmed by immunofluorescence analysis, as no cellular toxicity or significant changes in cellular morphology were seen according to the literature [28,30,35]. 

The presence of non-cytotoxic, signalling, protein-rich novel MBVs isolated from dBP was highlighted in this research; however, the work still holds some limitations. Because of the relative novelty of MBVs and their tissue-specificity, more in-depth characterisation should be performed to fully understand the biological potential of MBVs isolated from dBP, such as the characterisation of their surface markers and lipidic and nucleic acid content.

Given the results obtained with this research, demonstrating the rich bioactivity of MBVs, combined with the previously reported unique potential of MBVs for immunomodulatory and regenerative medicine applications, their ability to transfer their bioactive components and the advantage of their nanoscale nature, MBVs represent a promising and novel therapeutic aspect for regenerative medicine [27,28,29,30]. Research regarding the application of other EVs is more advanced and has already led to a number of commercial products and clinical trials for their application, in biomedical fields ranging from drug delivery to gene therapy, tissue regeneration, immunotherapy, cancer therapy, and diagnostics [48,79,80]. Because of the novelty of MBVs, the field of their applications is still immature; however, the inspiring results obtained with other types of EVs shed light on the possible future applications of MBVs and dECM biomaterials containing MBVs, opening a vast variety of possibilities and different areas of applications that could be studied.

In conclusion, nanovesicles isolated from a decellularised bovine pericardium extracellular matrix were identified, corresponding to the newly described MBVs. These MBVs were shown to carry a rich and bioactive cargo, including a tissue-specific set of proteins, cytokines, chemokines, and growth factors, recapitulating some of the contents found in the parent dBP. Moreover, the MBVs were shown to be cytocompatible and to enhance cell viability. These results pave the way for future studies on MBVs from dBP for regenerative medicine applications, including the assessment of their immunomodulatory, angiogenic, stem cell differentiation, and wound healing potentials.

## 4. Materials and Methods

### 4.1. Matrix-Bound Nanovesicles Isolation

Decellularised bovine pericardium extracellular matrix (dBP) was kindly provided by Tissuegraft Srl (Alessandria, Italy) (Italian patent number 102020000007567, patented on 29 April 2022; International patent number PCT/IB2021/052779 submitted on 2 April 2021). The dBP was lyophilised and ground to obtain a homogeneous powder. To extract MBVs, the dBP powder was enzymatically digested [10 mg of dry weight dBP/mL] by suspending it in a solution of Collagenase Type I [0.1 mg/mL] (Sigma-Aldrich, C0130, Milan, Italy), prepared in a Tris-HCl [50 mM, pH 7.4], NaCl [200 mM], and CaCl_2_ [5 mM] buffer. Enzymatic digestion in the Collagenase Type I solution was performed for 24 h at 37 °C under constant rotation. The digested dBP was centrifuged at 100× *g* for 5 min to remove any matrix residuals. The supernatant was collected and subjected to 10,000× *g* centrifugation for 90 min at 4 °C. The supernatant was collected and then ultracentrifuged at 100,000× *g* for 70 min at 4 °C (Optima™ LE-80K ultracentrifuge, Beckman Coulter, Brea, CA, USA) to isolate MBVs. The supernatant was discarded and the remaining pellet, containing MBVs, was then resuspended in particle-free PBS [100 mg of dry weight dBP/mL] and filtered with 0.22 μm pore filters (Sartorius, 17823-K, Bohemia, NY, USA). Samples diluted in PBS were stored at −20 °C until further use.

### 4.2. Nanoparticle Tracking Analysis

dBP MBVs’ size and concentration were determined using nanoparticle tracking assay (NTA). Briefly, resuspended MBVs were resuspended in particle-free PBS and analysed using a NanoSight N300 instrument (Malvern Panalytical, Malvern, UK). The size distribution of MBVs was determined by measuring the rate of Brownian motion with three video replicates. MBV size was represented as the mode ± SE (standard error) of the distribution; instead, MBV concentration was described as the mean MBVs/mL ± SE. 

### 4.3. Transmission Electron Microscopy

MBV sample droplets (1.9 × 10^8^ MBVs/mL) were placed on carbon-coated grids for 10 min, then fixed with 4% formaldehyde for 30 min. After washing the grids with distilled water and drying them, a post-fixation with 4% osmium tetroxide for 10 min at RT was performed, followed by washing the grids with distilled water and staining with 2% uranyl acetate for 10 min at RT. The grids were dried and then images were acquired at 120 kV with a Talos L120C G2 Transmission Electron Microscope (Thermo Fisher Scientific, Waltham, MA, USA).

### 4.4. Protein Content Characterisation

#### 4.4.1. Protein Extraction and Quantification

For SDS-PAGE and cytokine antibody array, proteins were extracted from MBVs according to an adaptation of Subedi et al.’s method [81]. Briefly, samples were lysed in radioimmunoprecipitation assay (RIPA) buffer for 30 min in ice, then dissociated mechanically using a gentleMACS™ Dissociator (Miltenyi Biotec, Gaithersburg, MD, USA). After sample lysis, protein concentration was determined using a bicinchoninic acid (BCA) protein assay kit (Novagen^®^, BCA Protein Assay Kit, 71285-3, Madison, WI, USA). For control, in both the SDS-PAGE and cytokine antibody array, a hydrogel derived from the enzymatic solubilisation of dBP was used (dBP gel) and also lysed as previously described.

#### 4.4.2. SDS-PAGE and Silver Staining

For SDS-PAGE, 1 μg protein extracts of MBVs were suspended in Laemmli Sample Buffer (Sigma-Aldrich, Milan, Italy) and loaded in a 10% acrylamide solution (40%) (ITW Reagents, A4989, Monza, Italy). The gels ran with a Mini-PROTEAN^®^ electrophoresis module (Bio-Rad, Hercules, CA, USA) at 100 mV in running buffer, composed of 0.1% SDS, 25 mM Trizma^®^ base (Sigma-Aldrich, T1503, Milan, Italy), and 192 mM glycine. After SDS-PAGE, silver staining was performed using the Pierce™ Silver Stain Kit (Thermo Fisher Scientific, Milan, Italy), according to the producer’s protocol. Gel images were acquired using a transilluminator ChemiDoc (Bio-Rad, Hercules, CA, USA). The experiment was performed in triplicate.

#### 4.4.3. Cytokine Antibody Array

Bovine Cytokine Array (RayBiotech, AAB-CYT-1, Peachtree Corners, GA, USA) was used, according to the manufacturer’s instructions, to evaluate the presence of 30 different cytokines in MBVs’ protein extracts. Briefly, capture antibodies were supplied arrayed/spotted on a membrane, with each pair of spots representing a different analyte, and were as follows: aFGF (FGF-1), Angiopoietin-1, bFGF, CD40 Ligand (TNFSF5), Decorin, GASP-1 (WFIKKNRP), IFN-alpha, IFN-beta, IFN-gamma, IGF-1, IL-1 alpha (IL-1 F1), IL-1 beta (IL-1 F2), IL-36 Ra (IL-1 F5), IL-10, IL-13, IL-15, IL-17A, IL-18, IL-2, IL-21, IL-4, IP-10 (CXCL10), LIF, MCP-1 (CCL2), MIG (CXCL9), MIP-1 beta (CCL4), NCAM-1 (CD56), RANTES (CCL5), TNF-alpha, and VEGF-A. The order of the membrane’s spots is shown in Table 2. A total of 50 μg/mL of the sample was used according to the producer’s protocol. The membranes were then imaged at ChemiDoc (Bio-Rad, Hercules, CA, USA) for chemiluminescence detection. Experiments were performed in triplicate.

#### 4.4.4. Protein Mass Spectrometry

MBV samples were frozen at −80 °C immediately after isolation for Liquid Chromatography with tandem mass spectrometry (LC-MS/MS). The samples were lysed with RIPA buffer and sonicated. Proteins were then precipitated with cold acetone and resuspended. Proteins were reduced in 25 µL of 100 mM NH_4_HCO_3_ with 2.5 μL of 200 mM DTT (Merck, Milan, Italy) at 60 °C for 45 min and next alkylated with 10 μL 200 mM iodoacetamide (Merck, Milan, Italy) for 1 h at room temperature in dark conditions. Iodoacetamide excess was removed by the addition of 200 mM DTT. The digests were dried via Speed Vacuum and then desalted [82]. Digested peptides were analysed on an Ultimate 3000 RSLC nano coupled directly to an Orbitrap Exploris 480 with a High-Field Asymmetric Waveform Ion Mobility Spectrometry System (FAIMSpro) (Thermo Fisher Scientific, Milan, Italy). Samples were injected onto a reversed-phase C18 column (15 cm × 75 µm i.d., Thermo Fisher Scientific, Milan, Italy) and eluted with a gradient of 6% to 95% mobile phase B over 40 min by applying a flow rate of 500 mL/min, followed by equilibration with 6% mobile phase B for 1 min. The acquisition time of one sample was 41 min and the total recording of the MS spectra was carried out in positive resolution with a high voltage of 2500 V and the FAIMS interface in standard resolution, with a CV of −45 V. The acquisition was performed in data-independent mode (DIA): precursor mass range was set between 400 and 900, isolation window of 8 *m*/*z*, window overlap of 1 *m*/*z*, HCD collision energy of 27%, orbitrap resolution of 30,000, and RF Lens at 50%. The normalised AGC target was set to 1000, the maximum injection time was 25 ms, and the microscan was 1. For DIA data processing, DIA-NN (version 1.8.1) was used: the identification was performed with “library-free search” and “deep learning-based spectra, RTs and IMs prediction” enabled. The enzyme was set to Trypsin/P; precursors of charge state 1–4, peptide lengths 7–30, and precursor *m*/*z* 400–900 were considered with a maximum of two missed cleavages. Carbamidomethylation on C was set as fixed modification and Oxidation on M was set as variable modification, using a maximum of two variable modifications per peptide. FDR was set to 1%.

### 4.5. Cytocompatibility

#### 4.5.1. Cell Culture

Normal human dermal fibroblast cells (NHDFs) (Lonza NHDF-Ad CC-2511, Basel, Switzerland) were used for cell viability assay and immunofluorescence. Cells were cultured in 75 cm^2^ flasks, maintained in Dulbecco’s Modified Eagle Medium (DMEM) (Gibco 21068-028, Milan, Italy), supplemented with 5 mM glutamine (Sigma-Aldrich 1294808, Milan, Italy), 1% penicillin, streptomycin and amphotericin-B (PSF) (Euroclone, Milan, Italy), and 10% foetal bovine serum (FBS) (Gibco, Milan, Italy). Cells were used up to the 6th passage.

#### 4.5.2. Cell Viability

MBV cytocompatibility was tested using MTT assay (MerckMillipore, Darmstadt, Germany). Cells were seeded in 48-well plates at a concentration of 8000 cells/well. After 24 h, to ensure adhesion, the culture medium was replaced with MBV-containing medium; specifically, MBVs previously diluted in PBS at different concentrations of 9 × 10^7^ MBVs/mL, 3.6 × 10^7^ MBVs/mL, and 1.8 × 10^7^ MBVs/mL. A total of 500 μL of PBS containing MBVs was placed in each well, and 500 μL of culture medium was added. In order to obtain a representative control, medium/PBS in a ratio of 1:1 was used. For a positive cell death control, 2.5% dimethyl sulfoxide (DMSO, Sigma-Aldrich, Milan, Italy) was used. At time points of 1 and 3 days after MBV addition to cells, MTT assay was performed by discarding culture media and adding DMEM 10% FBS supplemented with 0.5 mg/mL MTT (Sigma-Aldrich, Italy). After 4 h, media were discarded and 100 μL of DMSO was added. The 570 nm absorbance was read using Victor4X Multilabel Plate Reader (Perkin Elmer, Milan, Italy) and data were analysed in Excel (Microsoft, Redmond, WA, USA). Experiments were performed in triplicate.

#### 4.5.3. Immunofluorescence

MBVs’ effect on NHDF cell morphology was evaluated using phalloidin-DAPI immunofluorescence staining. Cells were seeded in 24-well plates over microscope glass slides, at a concentration of 14000 cells/well. After 24 h, the culture medium was replaced with MBV-containing medium; specifically, MBVs previously diluted in PBS at different concentrations of 9 × 10^7^ MBVs/mL, 3.6 × 10^7^ MBVs/mL, and 1.8 × 10^7^ MBVs/mL. A total of 750 μL of PBS containing MBVs was placed in each well, and 750 μL of culture medium was added. In order to obtain a representative control, a medium/PBS in a ratio of 1:1 was used. At time points of 1 and 3 days after MBVs were added to cells, samples were fixed using 4% formaldehyde for 1 h at room temperature. Phalloidin, Fluorescein Isothiocyanate Labeled (Sigma-Aldrich, P5282, Milan, Italy) was placed over the samples and incubated at 37 °C for 45 min. Nuclei were stained with 1 μg/mL DAPI (Sigma-Aldrich, Milan, Italy) for 1 min. Stained cells were observed using a fluorescence microscope (Leica DM700, Wetzlar, Germany), and images were acquired via Leica software LAS V4.7 (Leica, Wetzlar, Germany). Experiments were performed in triplicate.

## Figures and Tables

**Table 1:** Proteins found in MBV.1 and MBV.2 from LC-MS/MS. ✔: proteins represented in the MBV batch.

Protein	MBV.1	MBV.2
Total	45	23
Transthyretin	✔	
Keratocan	✔	
Prothrombin	✔	
Protein AMBP	✔	
Collagen alpha-1(I) chain	✔	✔
Collagen alpha-2(I) chain	✔	✔
Fibrinogen beta chain	✔	
Albumin	✔	✔
Annexin A2	✔	✔
Fibronectin	✔	✔
Heat shock 70 kDa protein 1-like	✔	✔
Galectin-1	✔	
Alpha-2-HS-glycoprotein	✔	✔
Apolipoprotein A-I	✔	
Mimecan	✔	✔
Decorin	✔	✔
Protein-lysine 6-oxidase	✔	
Alpha-1-antiproteinase	✔	
Vimentin	✔	✔
Transforming growth factor-beta-induced protein	✔	
Actin	✔	✔
Ras-related protein Rap-1b	✔	
Histone H4	✔	✔
14-3-3 protein zeta/delta	✔	
Elongation factor 1-alpha 1	✔	
Tubulin alpha-4A chain	✔	
Fibrillin-1	✔	✔
Endoplasmic reticulum chaperone BiP	✔	
Myosin-10	✔	
Latent-transforming growth factor beta-binding protein 2	✔	
Complement component C6	✔	✔
Tubulin beta-5 chain	✔	✔
Complement C3	✔	
Thrombospondin-4	✔	
Gelsolin	✔	
Heat shock protein beta-1	✔	
Asporin	✔	✔
Fibulin-5	✔	✔
Tropomyosin alpha-3 chain	✔	
Small ribosomal subunit protein eS12	✔	
Heat shock protein HSP 90-beta	✔	
Alpha-2-macroglobulin	✔	
Junction plakoglobin	✔	
Prolargin	✔	✔
Myocilin	✔	
Collagen alpha-1(III) chain		✔
LIM and SH3 domain protein 1		✔
Fibrinogen gamma-B chain		✔
Cadherin-13		✔
Poly(rC)-binding protein 1		✔

**Table 2:** The order of the cytokine spots on the Bovine Cytokine Array.

POS	POS	NEG	NEG	aFGF	Angiopoietin-1	bFGF	CD40 Ligand (TNFSF5)	Decorin	GASP-1 (WFIKKNRP)	IFN alpha	IFN beta
POS	POS	NEG	NEG	aFGF	Angiopoietin-1	bFGF	CD40 Ligand (TNFSF5)	Decorin	GASP-1 (WFIKKNRP)	IFN alpha	IFN beta
IFN-gamma	IGF-1	IL-1 alpha (IL-1 F1)	IL-1 beta (IL-1 F2)	IL-36 Ra (IL-1 F5)	IL-10	IL-13	IL-15	IL-17A	IL-18	IL-2	IL-21
IFN-gamma	IGF-1	IL-1 alpha (IL-1 F1)	IL-1 beta (IL-1 F2)	IL-36 Ra (IL-1 F5)	IL-10	IL-13	IL-15	IL-17A	IL-18	IL-2	IL-21
IL-4	IP-10 (CXCL10)	LIF	MCP-1 (CCL2)	MIG (CXCL9)	MIP-1 beta (CCL4)	NCAM-1 (CD56)	RANTES (CCL5)	TNF alpha	VEGF-A	BLANK	POS
IL-4	IP-10 (CXCL10)	LIF	MCP-1 (CCL2)	MIG (CXCL9)	MIP-1 beta (CCL4)	NCAM-1 (CD56)	RANTES (CCL5)	TNF alpha	VEGF-A	BLANK	POS

## Data Availability

Data are contained within the article.
